# Zinc supplementation and 60-day mortality in patients receiving total parenteral nutrition: a single-center experience

**DOI:** 10.3389/fnut.2026.1735455

**Published:** 2026-02-04

**Authors:** Mei-Yuan Liu, Chia-Yin Kuo, Hwung-Chung Lee, Jheng-Yan Wu

**Affiliations:** 1 Department of Nutrition, Chi Mei Medical Center, Tainan, Taiwan; 2 Department of Nutrition and Health Sciences, Chang Jung Christian University, Tainan, Taiwan; 3 Department of Hospitality Management, Southern Taiwan University of Science and Technology, Tainan, Taiwan; 4 Department of Nursing, Chi Mei Medical Center, Tainan, Taiwan; 5 Department of Public Health, College of Medicine, National Cheng Kung University, Tainan, Taiwan

**Keywords:** all-cause mortality, retrospective study, total parenteral nutrition, trace element, zinc supplementation

## Abstract

**Objective:**

Zinc deficiency is common among patients receiving total parenteral nutrition (TPN) and may contribute to impaired wound healing, immune dysfunction, and adverse clinical outcomes. However, the impact of zinc supplementation on short-term survival remains unclear.

**Methods:**

We retrospectively reviewed adult patients who received TPN at a single center between January 2019 and October 2023. Patients were categorized according to their mean daily zinc dose: < 2 mg/day or ≥2 mg/day. Patients were classified into a standard zinc supplementation group receiving approximately 6.35 mg of elemental zinc per day and an augmented zinc supplementation group receiving approximately 7.7 mg per day. Baseline characteristics, nutritional indices, and biochemical parameters were compared. The primary outcome was 60-day all-cause mortality. Hazard ratios (HRs) and 95% confidence intervals (CIs) were estimated using Cox proportional hazards models.

**Results:**

A total of 1,037 patients were included (415 in the standard zinc supplementation group and 622 in the augmented zinc supplementation group). Baseline characteristics were comparable between groups (mean age 65 years; 60% male). The augmented zinc supplementation group had higher mean serum zinc concentrations (75.9 ± 22.0 μg/dl vs. 65.9 ± 27.5 μg/dL, *P* < 0.001). The 60-day mortality rate was significantly lower in the augmented zinc supplementation group (22.7% vs. 15.0%; HR = 0.66; 95% CI 0.50–0.88; *P* = 0.004). No major differences were found in caloric or protein adequacy, albumin, or C-reactive protein trends between groups.

**Conclusion:**

In this single-center retrospective cohort, higher zinc supplementation (≥2 mg/day) during TPN administration was associated with lower 60-day mortality. These findings highlight the potential clinical relevance of zinc dosing in TPN regimens and warrant prospective validation.

## Introduction

Zinc is an essential trace element that plays a fundamental role in numerous physiological processes, including immune regulation, protein synthesis, cellular proliferation, and wound healing (1, 2). In hospitalized patients, especially those requiring total parenteral nutrition (TPN), zinc deficiency is a frequent and clinically important problem (3, 4). Because these patients are unable to obtain nutrients through the gastrointestinal tract, their micronutrient supply depends entirely on parenteral formulations. Inadequate zinc intake during TPN can lead to delayed wound healing, impaired immune response, diarrhea, dermatitis, and increased susceptibility to infections, all of which can adversely affect recovery and survival (5–7).

Clinical guidelines recommend routine zinc supplementation for patients receiving TPN, with dose adjustments based on metabolic stress, gastrointestinal losses, and disease severity (2, 8–10). However, the optimal zinc dose in clinical practice remains controversial, particularly in surgical and critically ill populations (11).

Current ASPEN and ESPEN guidelines recommend approximately 2–5 mg of elemental zinc per day in standard parenteral nutrition formulations with higher doses required for patients with increased gastrointestinal losses or metabolic stress (2, 8–10). In our institution, additional zinc supplementation of at least 2 mg per day was initiated when serum concentrations were low or clinical demand was increased, consistent with these recommendations. Many hospitals, including ours, routinely add a standard dose of zinc sulfate to TPN solutions, but this amount may be insufficient for patients with increased metabolic demand, such as those undergoing major abdominal surgery or experiencing enteric fistulas or sepsis. Moreover, in real-world practice, the decision to provide additional zinc supplementation is often influenced by patient affordability, as higher doses are typically self-funded.

Previous studies have primarily focused on zinc deficiency and its biochemical or immunologic manifestations, whereas limited data have explored its association with hard clinical outcomes such as mortality. Observational studies have suggested that low serum zinc concentrations correlate with higher mortality and prolonged hospitalization, but evidence regarding the survival benefits of zinc repletion in TPN-dependent patients is scarce (12–17).

Since January 2019, our center has implemented routine monitoring of serum zinc levels in patients receiving TPN and adopted individualized supplementation strategies following guideline recommendations. This policy created a natural variation in zinc dosage among patients, providing a unique opportunity to evaluate its clinical impact. Therefore, this retrospective study aimed to investigate whether higher zinc supplementation (≥2 mg/day) during TPN administration is associated with improved short-term outcomes, particularly 60-day all-cause mortality, in hospitalized adults. The findings may help inform practical dosing strategies and reinforce the importance of trace element optimization in parenteral nutrition care.

## Methods

### Study design and population

This retrospective observational cohort study was conducted at a center. The study included adult patients (aged ≥18 years) who received TPN between January 1, 2019, and October 31, 2023. Clinical and biochemical data were retrieved from the institutional electronic medical record system. The study protocol was approved by the Chi Mei Medical Center Institutional Review Board (CMFHR112110), and the requirement for informed consent was waived due to the retrospective design and anonymized data handling.

### Inclusion and exclusion criteria

All hospitalized adults who received TPN during the study period were screened. Patients were eligible if they had available baseline serum zinc concentrations measured within the first week of TPN initiation. Exclusion criteria were (1) age below 18 years, (2) administration of compounded three-in-one parenteral nutrition solutions, or (3) missing baseline zinc data. Patients receiving commercially prepared three-in-one parenteral nutrition solutions were excluded because these formulations have fixed trace element content and do not permit individualized zinc dosing or accurate quantification of elemental zinc exposure. After applying these criteria, a total of 1,037 patients were included in the final analysis.

### Exposure definition

Patients were categorized into two groups according to their average daily elemental zinc dose received during TPN administration. In our institution, all standard TPN formulations contained a multi-trace element solution that provided approximately 5 mg of elemental zinc per day, consistent with institutional and guideline recommendations. In addition, one ampoule of zinc sulfate (1.35 mg elemental zinc) was routinely added to each TPN bag as part of standard practice. Patients who received only this standard regimen were classified as the standard zinc supplementation group (< 2 mg/day additional zinc). In contrast, patients whose serum zinc concentrations were below the reference range or who had higher metabolic or gastrointestinal losses received two ampoules of zinc sulfate daily (approximately 2.7 mg elemental zinc) and were defined as the augmented zinc supplementation group (≥2 mg/day additional zinc).

Standard TPN formulations provided approximately 6.35 mg of elemental zinc per day. Patients requiring additional supplementation received one extra ampoule of zinc sulfate resulting in a total intake of approximately 7.7 mg per day. Groups were therefore defined based on supplemental dosing and are referred to as the standard zinc supplementation group and the augmented zinc supplementation group. All patients received a standardized amount of elemental zinc from routine trace element formulations. The exposure definition was therefore based on additional zinc supplementation beyond this baseline to reflect differences in clinical supplementation strategies.

All TPN formulations were compounded by hospital pharmacists and administered under the supervision of the multidisciplinary nutrition support team. Serum zinc concentrations were routinely measured on a weekly basis during TPN therapy to ensure adequacy and guide individualized supplementation according to guidelines.

### Data collection

Demographic and clinical characteristics were collected, including age, sex, body mass index (BMI), length of hospital stay, surgical status, and presence of malignancy. Nutritional indicators included the Subjective Global Assessment (SGA), daily caloric and protein intake, and serum biochemical parameters (albumin, prealbumin, C-reactive protein [CRP], and white blood cell count). Baseline laboratory values were obtained before TPN initiation, and follow-up data were recorded weekly thereafter. Although serum zinc concentrations were measured weekly for clinical monitoring, only the baseline value obtained prior to or within the first week of TPN initiation was used in the analysis. Time updated zinc values were not incorporated because follow up measurements were not available at consistent intervals for all patients.

### Outcome measures

The primary outcome was 60-day all-cause mortality following TPN initiation. Secondary outcomes included changes in serum zinc concentration and biochemical markers of nutritional and inflammatory status during hospitalization.

### Statistical analysis

Continuous variables were expressed as mean ± standard deviation (SD) and compared using the *t*-test. Categorical variables were reported as frequencies and percentages and compared using the chi-square or Fisher's exact test, as appropriate. A Cox proportional hazards model was then used to estimate the association between zinc dosage category and 60-day mortality, adjusting for clinically relevant covariates. Results were expressed as hazard ratios (HRs) with 95% confidence intervals (CIs). Missing data were handled using a complete case approach. Patients without baseline serum zinc measurements were excluded during cohort assembly, and multivariable analyses were restricted to participants with available covariate data. No imputation methods were applied. All analyses were conducted using SPSS version 25.0 (IBM Corp., Armonk, NY, USA), and a two-tailed *P* value < 0.05 was considered statistically significant.

## Results

### Baseline characteristics

The mean total daily zinc intake was approximately 6.35 mg in the standard zinc supplementation group and approximately 7.7 mg in the augmented zinc supplementation group. A total of 1,037 patients who received TPN between January 2019 and October 2023 were included in the final analysis, comprising 622 (60%) in the augmented zinc supplementation group (≥2 mg/day additional zinc) and 415 (40%) in the standard zinc supplementation group (< 2 mg/day additional zinc). Baseline demographic and clinical characteristics were broadly comparable between groups (Table 1). The mean age of the study population was 65 years, and approximately 60% were male. Mean BMI was similar between the standard zinc supplementation and augmented zinc supplementation groups (22.9 ± 8.5 vs. 23.3 ± 11.0 kg/m^2^, *P* = 0.55). The prevalence of malignancy was 64% and 61% in the standard zinc supplementation and augmented zinc supplementation groups, respectively (*P* = 0.39). The proportion of surgical patients was higher in the standard zinc supplementation group (80%) compared with the augmented zinc supplementation group (71%, *P* < 0.001).

**Table 1:** Baseline demographic, clinical, and biochemical characteristics of patients receiving total parenteral nutrition according to zinc supplementation level.

**Variable**	**Standard zinc supplementation (< 2 mg/day)**	**Augmented zinc supplementation (≥2 mg/day)**	***P* value**
No. of patients	415	622	
Male, *n* (%)	249 (60.0)	373 (60.0)	0.94
Age, years	64.8 ± 13.9	65.3 ± 13.9	0.58
BMI, kg/m^2^	22.9 ± 8.5	23.3 ± 11.0	0.55
Surgical patient, *n* (%)	332 (80.0)	440 (71.0)	**<** **0.001**
Malignancy, *n* (%)	263 (64.0)	376 (61.0)	0.39
Serum zinc, μg/dL	56.0 ± 20.1	63.1 ± 19.2	**<** **0.001**
Albumin, g/dL	2.7 ± 0.7	2.7 ± 0.7	0.56
Prealbumin, mg/dL	13.2 ± 7.1	14.4 ± 8.0	0.022
WBC, × 103/μL	11.4 ± 9.0	10.3 ± 6.1	0.024
Length of stay, days	41.1 ± 27.7	42.3 ± 31.5	0.53

Values are presented as mean ± SD or number (%) as appropriate. BMI, body mass index; WBC, white blood cell count.

Bold values indicate statistically significant differences (*p* < 0.05).

### Biochemical and nutritional findings

At baseline, serum zinc concentrations were significantly higher in the augmented zinc supplementation group (63.1 ± 19.2 μg/dL) than in the standard zinc supplementation group (56.0 ± 20.1 μg/dL, *P* < 0.001). Other nutritional indicators such as serum albumin (2.7 ± 0.7 vs. 2.7 ± 0.7 mg/dL, *P* = 0.56) were comparable between groups, whereas prealbumin levels were slightly higher in the augmented zinc supplementation group (14.4 ± 8.0 vs. 13.2 ± 7.1 mg/dL, *P* = 0.022). The augmented zinc supplementation group also showed a lower mean white blood cell count (10.3 ± 6.1 × 103/μl vs. 11.4 ± 9.0 × 103/μl, *P* = 0.024).

During follow-up, both groups demonstrated gradual increases in serum zinc concentrations after 28 days of TPN; however, the mean zinc level at day 28 showed a minor trend toward higher levels in the augmented zinc supplementation group, although the difference did not reach statistical significance compared with the standard zinc supplementation group (75.9 ± 22.0 μg/dL vs. 65.9 ± 27.5 μg/dL, *P* = 0.087). No significant between-group differences were observed in CRP, albumin, or prealbumin levels at day 28. The mean hospital length of stay was similar between groups (42.3 ± 31.5 vs. 41.1 ± 27.7 days, *P* = 0.53).

### Sixty-day mortality outcomes

**Figure 1:**
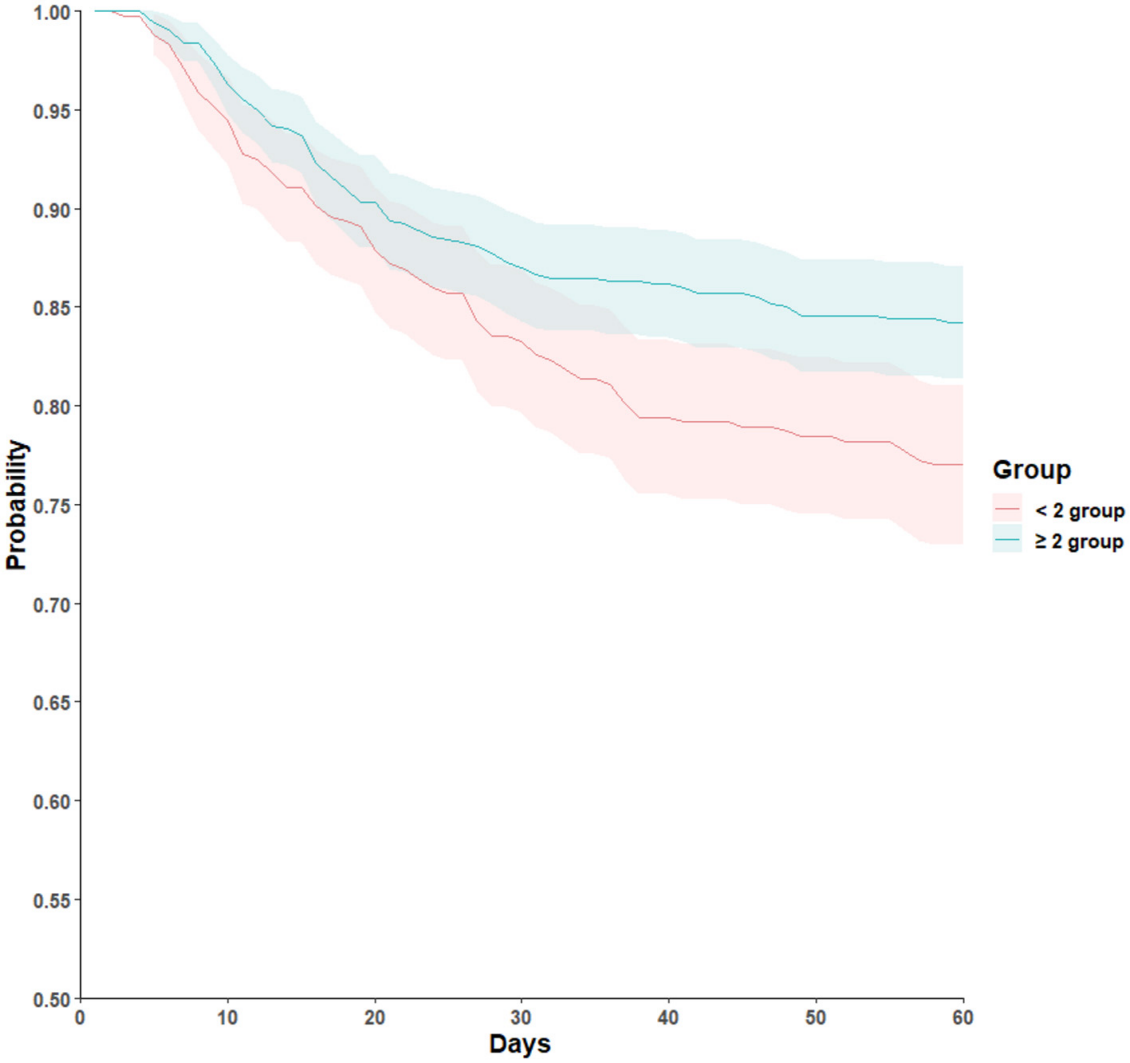
Kaplan-Meier survival curves for 60-day all-cause mortality according to zinc supplementation level during total parenteral nutrition. Kaplan-Meier curves depict unadjusted survival and should be interpreted in conjunction with adjusted Cox regression results.

In survival analyses, patients in the augmented zinc supplementation group had a significantly lower 60-day all-cause mortality rate compared with those in the standard zinc supplementation group (Figure 1). After adjustment for age, sex, surgical status, malignancy, and SGA category, the association between higher zinc supplementation and improved 60-day survival remained significant (22.7% vs. 15.0%; HR 0.66; 95% CI 0.50–0.88; *P* = 0.004; Table 2). Other variables independently associated with increased mortality included surgical intervention (HR 1.65; 95% CI 1.23–2.23; *P* = 0.001) and poorer nutritional status (SGA category A vs. C, HR 2.99; 95% CI 1.69–5.29; *P* < 0.001).

**Table 2:** Cox proportional hazards model of factors associated with 60-day all-cause mortality among patients receiving total parenteral nutrition.

**Variable**	**Relative risk (95% CI)**	***P* value**
Augmented zinc supplementation (≥ 2 mg/day)	0.66 (0.50–0.88)	**0.004**
Male sex	0.99 (0.74–1.32)	0.92
Surgical procedure	1.65 (1.23–2.23)	**0.001**
Malignancy	1.23 (0.93–1.64)	0.15
ICU vs. medical ward	3.85 (2.34–6.34)	**<** **0.001**
SGA A vs. C	2.99 (1.69–5.29)	**<** **0.001**
Age (per year)	1.00 (0.99–1.01)	0.52
BMI (per kg/m^2^)	1.00 (0.99–1.01)	0.99

CI, confidence interval; ICU, intensive care unit; SGA, subjective global assessment; BMI, body mass index.

Bold values indicate statistically significant differences (*p* < 0.05).

## Discussion

In this retrospective single-center study, we observed that patients receiving TPN with higher zinc supplementation (≥2 mg/day additional zinc) demonstrated a significantly lower 60-day all-cause mortality compared with those who received the standard regimen. This association remained significant after adjusting for potential confounders, including age, sex, surgical status, malignancy, and nutritional category. Although this observational design precludes causal inference, our findings highlight the potential clinical relevance of adequate zinc supplementation in association with short-term survival among patients dependent on TPN.

Zinc deficiency is widely recognized in patients requiring TPN, particularly those with increased metabolic stress, gastrointestinal losses, or postoperative complications (18–21). As zinc plays essential roles in protein synthesis, wound healing, and immune regulation, insufficient supplementation during TPN can exacerbate catabolic stress and delay recovery (22–24). Both guidelines emphasize individualized zinc supplementation, especially in patients with high output fistulas, diarrhea, or sepsis, where losses are substantially increased (2, 8–10). However, despite guideline recommendations, under-supplementation remains common in clinical practice due to limited awareness or financial constraints, as higher doses are often self-funded in some healthcare systems, including ours.

The present findings align with previous research showing that low serum zinc levels are associated with prolonged hospitalization, impaired immune function, and increased mortality in critically ill or malnourished populations (15, 25, 26). For example, several observational studies have reported that zinc deficiency correlates with higher risk of infection, delayed wound healing, and longer duration of mechanical ventilation in intensive care units (27, 28). Moreover, zinc repletion has been shown to modulate inflammatory pathways by reducing oxidative stress and downregulating proinflammatory cytokine expression (29–31). In the context of TPN, maintaining adequate zinc levels may therefore support mucosal barrier integrity and immune competence, both of which are crucial in preventing nosocomial complications and sepsis-related mortality.

Interestingly, our study showed that despite similar caloric and protein adequacy between groups, higher zinc supplementation was associated with improved survival, suggesting that micronutrient optimization may be associated with outcomes beyond macronutrient delivery. This finding supports the notion that trace element adequacy is a vital yet often underappreciated component of parenteral nutrition therapy. Furthermore, serum zinc levels in our cohort gradually increased during TPN administration, confirming that supplementation effectively corrected biochemical deficiency. Nonetheless, the mean baseline serum zinc concentrations in both groups were below the lower limit of normal, underscoring the high prevalence of zinc deficiency among hospitalized patients receiving TPN.

In this observational study, zinc supplementation was administered based on clinical judgment, including low serum zinc concentrations and conditions associated with increased metabolic demand or gastrointestinal loss. As a result, confounding by indication is likely. The high supplementation group demonstrated lower baseline zinc levels and a higher proportion of non-surgical cases which may indicate greater clinical complexity. Although multivariable models adjusted for several prognostic factors residual confounding cannot be excluded. Unmeasured variables such as comorbidity burden or clinical severity may have influenced both treatment allocation and mortality risk. Notably the high supplementation group had lower adjusted mortality despite potential baseline disadvantage which is consistent with but does not prove a beneficial effect of zinc supplementation. Future studies using randomized allocation stratified dosing or causal inference designs are needed to establish whether correcting zinc deficiency can causally improve survival.

Several mechanisms may explain the observed relationship between higher zinc supplementation and improved survival. Zinc acts as a cofactor for more than 300 enzymes involved in DNA synthesis, antioxidant defense, and cell-mediated immunity (32). In catabolic or inflammatory states, redistribution of zinc from plasma to tissues can result in apparent hypozincemia, which may further impair host defense and tissue repair (33). Adequate zinc supplementation during TPN could therefore help restore normal physiological function and mitigate the deleterious effects of systemic inflammation. Although zinc may exert anti inflammatory effects, no significant differences in CRP were observed between groups. This may reflect the dominant influence of underlying illness, surgical stress, and infection on CRP values, which could mask micronutrient related effects. It is also possible that the anti inflammatory actions of zinc are mediated through mechanisms not captured by conventional markers such as CRP, including modulation of cytokine signaling and oxidative stress pathways (34). However, serum zinc concentrations are influenced by the acute phase response and may not accurately reflect functional zinc status, particularly in critically ill patients. Accordingly, baseline zinc values should be interpreted descriptively rather than causally.

This study has several limitations. First, the retrospective design precludes causal inference and residual confounding remains possible despite multivariable adjustment. Zinc supplementation was prescribed based on clinical judgment rather than random allocation, introducing confounding by indication, as patients with greater clinical severity or metabolic demand were more likely to receive higher supplementation. Although pragmatic proxies of illness severity, including ICU admission, surgical status, malignancy, and nutritional status, were adjusted for, detailed severity scores such as APACHE II or SOFA and comprehensive organ failure measures were not uniformly available, and residual confounding cannot be excluded. Second, serum zinc concentrations may not accurately reflect functional zinc status because they are influenced by the acute phase response, particularly in critically ill patients, and were therefore interpreted descriptively rather than causally. Third, because all patients received zinc as part of standard TPN formulations, this study compared standard vs. augmented zinc supplementation strategies rather than total zinc dose as a continuous exposure, and dose response relationships could not be formally assessed. Early mortality after TPN initiation may also have limited exposure duration in some patients, potentially biasing observed survival differences. In addition, cumulative zinc exposure and detailed duration of supplementation were not modeled. Accordingly, the analysis reflects differences in supplementation strategies rather than total zinc dose over time. Finally, this was a single center study, which may limit generalizability. Despite these limitations, our findings contribute to the growing body of evidence emphasizing the importance of trace element optimization in TPN care. Given the observed association with survival, future prospective randomized controlled trials are warranted to determine the optimal zinc dose, evaluate long-term outcomes, and clarify whether targeted supplementation strategies can improve clinical recovery and cost-effectiveness.

## Conclusion

In conclusion, this single-center retrospective study demonstrated that higher zinc supplementation during TPN administration was associated with lower 60-day all-cause mortality. Ensuring adequate trace element replacement, particularly zinc, may represent an important consideration in the care of patients receiving parenteral nutrition.

## Data Availability

The original contributions presented in the study are included in the article/supplementary material, further inquiries can be directed to the corresponding author.

## References

[1] Ranasinghe P Pigera S Galappatthy P Katulanda P Constantine GR. Zinc and diabetes mellitus: understanding molecular mechanisms and clinical implications. Daru. (2015) 23:44. doi: 10.1186/s40199-015-0127-4 26381880 PMC4573932

[2] Berger MM Shenkin A Dizdar OS Amrein K Augsburger M Biesalski HK . ESPEN practical short micronutrient guideline. Clin Nutr. (2024) 43:825–57. doi: 10.1016/j.clnu.2024.01.030 38350290

[3] Jeejeebhoy K. Zinc: an essential trace element for parenteral nutrition. Gastroenterology. (2009) 137:S7–12. doi: 10.1053/j.gastro.2009.08.014 19874952

[4] Younoszai HD. Clinical zinc deficiency in total parenteral nutrition: zinc supplementation. J Parenter Enteral Nutrition. (1983) 7:72–4. doi: 10.1177/014860718300700172 6403736

[5] Singer P Blaser AR Berger MM Calder PC Casaer M Hiesmayr M . ESPEN practical and partially revised guideline: clinical nutrition in the intensive care unit. Clin Nutr. (2023) 42:1671–89. doi: 10.1016/j.clnu.2023.07.011 37517372

[6] Singer P Blaser AR Berger MM Alhazzani W Calder PC Casaer MP . ESPEN guideline on clinical nutrition in the intensive care unit. Clin Nutr. (2019) 38:48–79. doi: 10.1016/j.clnu.2018.08.037 30348463

[7] Berlana D. Parenteral nutrition overview. Nutrients. (2022) 14:4480. doi: 10.3390/nu14214480 36364743 PMC9659055

[8] Ayers P Adams S Boullata J Gervasio J Holcombe B Kraft MD . ASPEN parenteral nutrition safety consensus recommendations. J Parenter Enteral Nutr. (2014) 38:296–333. doi: 10.1177/0148607113511992 24280129

[9] Boullata JI Gilbert K Sacks G Labossiere RJ Crill C Goday P . ASPEN clinical guidelines: parenteral nutrition ordering, order review, compounding, labeling, and dispensing. J Parenter Enteral Nutr. (2014) 38:334–77. doi: 10.1177/0148607114521833 24531708

[10] Boullata JI Carrera AL Harvey L Escuro AA Hudson L Mays A . ASPEN safe practices for enteral nutrition therapy. J Parenter Enteral Nutr. (2017) 41:15–103. doi: 10.1177/0148607116673053 27815525

[11] Suruli PK Rangappa P Jacob I Rao K Shivashanker S. Zinc deficiency in critically ill patients: impact on clinical outcome. Cureus. (2024) 16:e61690. doi: 10.7759/cureus.61690 38975455 PMC11224045

[12] Perks P Huynh E Kaluza K Boullata JI. Advances in trace element supplementation for parenteral nutrition. Nutrients. (2022) 14:1770. doi: 10.3390/nu14091770 35565737 PMC9105959

[13] Wu JY Wu YJ Liu MY Hsu WH Tsai YW Liu TH . Clinical outcomes in diabetic patients with zinc deficiency: a multi-institutional population-based study. J Am Nutr Assoc. (2025) 44:521–8. doi: 10.1080/27697061.2025.2461215 39908138

[14] Lin YM Tu WL Hung KC Yu T Wu JY Lai CC. Mortality and cardiorenal outcomes among heart failure patients with zinc deficiency: a multicenter retrospective cohort study of 8,290 patients. Front Nutr. (2025) 12:1589907. doi: 10.3389/fnut.2025.1589907 40357034 PMC12066520

[15] Hung LW Liu MY Yu T Hung KC Tsai YW Lai CC . Zinc deficiency and post-acute outcomes in patients with COVID-19: a six-month retrospective cohort analysis of 3,726 patients. Cureus. (2024) 16:e71609. doi: 10.7759/cureus.71609 39553000 PMC11566094

[16] Wu JY Hsu WH Tsai YW Liu TH Huang PY Chuang MH . The association between zinc deficiency, and clinical outcomes of COVID-19. J Infect. (2023) 87:e63–e7. doi: 10.1016/j.jinf.2023.06.021 37393053

[17] Wu JY Liu TH Huang PY Tsai YW Lai CC. The effect of zinc on the outcome of patients with COVID-19: a systematic review and meta-analysis of randomized controlled trials. J Infect. (2023) 86:e142–e3. doi: 10.1016/j.jinf.2023.01.023 36693569 PMC9867826

[18] Stehle P Stoffel-Wagner B Kuhn KS. Parenteral trace element provision: recent clinical research and practical conclusions. Eur J Clin Nutr. (2016) 70:886–93. doi: 10.1038/ejcn.2016.53 27049031 PMC5399133

[19] Baddam S Maxfield L Shukla S Crane JS. Zinc Deficiency. Treasure Island, FL: StatPearls Publishing LLC (2025). 29630283

[20] Lin PH Sermersheim M Li H Lee PHU Steinberg SM Ma J. Zinc in wound healing modulation. Nutrients. (2017) 10:16. doi: 10.3390/nu10010016 29295546 PMC5793244

[21] Arribas Lopez E Zand N Ojo O Kochhar T. Systematic review and meta-analysis of the effect of zinc on wound healing. BMJ Nutr Prev Health. (2025) 8:e000952. doi: 10.1136/bmjnph-2024-000952 40771531 PMC12322555

[22] Ghaly P Iliopoulos J Ahmad M. The role of nutrition in wound healing: an overview. Br J Nurs. (2021) 30:S38–s42. doi: 10.12968/bjon.2021.30.5.S38 33733851

[23] Ju M Kim Y Seo KW. Role of nutrition in wound healing and nutritional recommendations for promotion of wound healing: a narrative review. Ann Clin Nutr Metab. (2023) 15:67–71. doi: 10.15747/ACNM.2023.15.3.67

[24] Jin D Wei X He Y Zhong L Lu H Lan J . The nutritional roles of zinc for immune system and COVID-19 patients. Front Nutr. (2024) 11:1385591. doi: 10.3389/fnut.2024.1385591 38706559 PMC11066294

[25] Chen IW Yu TS Lai YC Liu PH Chang YJ Wu JY . Impact of zinc deficiency on mortality risk in patients with chronic obstructive pulmonary disease: a retrospective analysis. Front Nutr. (2025) 12:1655272. doi: 10.3389/fnut.2025.1655272 41141262 PMC12549255

[26] Knoell DL Julian MW Bao S Besecker B Macre JE Leikauf GD . Zinc deficiency increases organ damage and mortality in a murine model of polymicrobial sepsis. Crit Care Med. (2009) 37:1380–8. doi: 10.1097/CCM.0b013e31819cefe4 19242332 PMC2905048

[27] Kavaliova H Yamano S Murahashi S Sato S Kawazoe Y Uemura E . Analysis of serum zinc level in patients with severe trauma. J Clin Biochem Nutr. (2025) 76:296–303. doi: 10.3164/jcbn.24-87 40510393 PMC12152239

[28] Chinni V El-Khoury J Perera M Bellomo R Jones D Bolton D . Zinc supplementation as an adjunct therapy for COVID-19: challenges and opportunities. Br J Clin Pharmacol. (2021) 87:3737–46. doi: 10.1111/bcp.14826 33742473 PMC8250380

[29] Schulz MT Rink L. Zinc deficiency as possible link between immunosenescence and age-related diseases. Immun Ageing. (2025) 22:19. doi: 10.1186/s12979-025-00511-1 40390089 PMC12087153

[30] Wessels I Maywald M Rink L. Zinc as a gatekeeper of immune function. Nutrients. (2017) 9:1286. doi: 10.3390/nu9121286 29186856 PMC5748737

[31] Briassoulis G Briassoulis P Ilia S Miliaraki M Briassouli E. The anti-oxidative, anti-inflammatory, anti-apoptotic, and anti-necroptotic role of zinc in COVID-19 and sepsis. Antioxidants. (2023) 12:1942. doi: 10.3390/antiox12111942 38001795 PMC10669546

[32] Stiles LI Ferrao K Mehta KJ. Role of zinc in health and disease. Clin Exp Med. (2024) 24:38. doi: 10.1007/s10238-024-01302-6 38367035 PMC10874324

[33] Prasad AS. Discovery of human zinc deficiency and studies in an experimental human model. Am J Clin Nutr. (1991) 53:403–12. doi: 10.1093/ajcn/53.2.403 1989405

[34] Sankova M Nikolenko V Oganesyan M Vinnik Y Gavryushova L Redina S . Zinc pathogenic importance in correcting immunity and restoring public health in the post-COVID period: an overview. Cytokine. (2024) 184:156761. doi: 10.1016/j.cyto.2024.156761 39307118

